# Coping with weight stigma: Validation of the Persian brief coping responses inventory with Iranian adolescents sample

**DOI:** 10.3389/fpsyg.2022.996052

**Published:** 2022-11-04

**Authors:** Leila Kargari Padar, Ali Asghar Asgharnejad Farid, Fahimeh Fathali Lavasani, Hojjatollah Farahani, Banafsheh Gharaei

**Affiliations:** ^1^Department of Clinical Psychology, School of Behavioral Sciences and Mental Health (Tehran Institute of Psychiatry), Iran University of Medical Sciences, Tehran, Iran; ^2^Department of Psychology, Tarbiat Modares University, Tehran, Iran

**Keywords:** coping, obesity, BCRI, weight stigma, psychometrics

## Abstract

Individuals who are overweight or obese encounter frequent weight-related stigma experiences, which are associated with negative health outcomes. In this regard, the Brief Coping Responses Inventory (BCRI) was developed as a measure of core coping responses to weight stigma, with 10 items loading on two subscales of reappraisal and Disengagement coping. The current study aimed to examine the psychometric properties of the Persian BCRI with 253 Iranian school-attending youth (*M_age_* = 15.38, *SD* ± 0.50; 53% female) who had BMI score over 30. The results of the confirmatory factor analysis supported the originally proposed two-factor model (RMSEA = 0.028; CFI = 0.996; TLI = 0.994), which yielded acceptable internal consistency based on various reliability measures such as Cronbach’s alpha coefficients (0.79 and 0.88 for reappraisal and disengagement factors, respectively) and demonstrated the expected convergent and divergent associations with external correlates of interest (e.g., anxiety, depression, and body dissatisfaction scores). This study was also the first one worldwide to examine and report that the originally proposed two-factor model of the BCRI is invariant across gender groups, which allows for examining mean differences in BCRI scores across gender groups. Overall, our results indicated that the BCRI is a valid and reliable measure with a solid factor structure, which could be used to examine the coping reactions to weigh related stigma experiences among youth samples in Iran. Our results may encourage future studies on the psychometrics of the BCRI with other Iranian samples (e.g., university students, community, and clinical samples).

## Introduction

Weight-based stigmatization is a pervasive concern associated with adverse health outcomes such as low levels of self-esteem, depression, anxiety, body dissatisfaction, and weight gain over time for individuals targeted (Jackson et al., 2014; Pearl and Puhl, 2016). People with obesity and overweight issues are negatively labeled as lazy, lacking willpower and self-control, lacking the motivation to enhance their health, and are criticized for their weight (Puhl and Heuer, 2009). Reinforcement of these negative attitudes in various societal environments, along with a lack of organized measures to contest these visions, has resulted in social disapproval, stigma, and unjust behaviors towards individuals with obesity (Pearl, 2018). While weight stigma is recognized as a stressful experience, there is very little information on how people cope with these stigmatizing experiences (Hayward et al., 2017). A review by Puhl and Brownell (2003) recognized several potential coping responses that individuals resort to in dealing with weight stigma. Accordingly, victims of weight stigma may react by blending to weight-based stereotypes, utilizing self-protection procedures (e.g., attributing adverse feedback to the prejudiced ideas of others), offsetting weight-related negativity by becoming skilled in other activities (e.g., enhancing their social skillfulness to become more admirable), attributing their obesity and weight-related conducts to factors beyond their control (e.g., medication), negotiating their identity (e.g., rejecting their weight-based identity or reducing its significance in frightening circumstances), facing the perpetrator of the stigma, engaging in social activism, clinging to avoidance or disengagement procedures (e.g., evading areas where stigma is possible to happen), engaging in communal coping or endeavoring to lose weight. These procedures may be demonstrated in various ways, and diverse individual and situational elements determine the degree to which these reactions are used.

All these taken into account, it is important to develop and validate measures to assess individuals’ coping reactions to weight stigma. In this vein, Myers and Rosen developed Coping Responses Inventory (CRI) with 99 items as a counterpart tool to their Stigmatizing Situations Inventory (SSI; Myers and Rosen, 1999). The CRI has 21 subfactors and measures a broad spectrum of coping responses individuals implement as reactions to weight stigma. These reactions could be categorized as cognitive (self-talk, reappraisal) and behavioral (pursuing social support, confronting the perpetrator); and positive (self-love) or negative (negative self-talk, isolating oneself). Myers and Rosen found that three CRI subscales, i.e., pessimistic self-talk, cry/separate myself, and evade or leave the circumstances, were significantly correlated with adverse psychological outcomes. However, they could not recognize any ‘adaptive’ coping responses associated with positive psychological outcomes (Myers and Rosen, 1999). Notwithstanding, in another study, using a revised version of the CRI (with an adjusted response scale), Puhl and Brownell (2003) examined coping responses to weight stigma with a mixed gender sample from a weight-loss support institution and discovered some evidence of ‘adaptive’ coping styles (pursuing social support, optimistic self-talk). Nonetheless, considering the length of the CRI, it might not be practical for research purposes, which might explain why very rare studies have explored how individuals cope with weight stigma.

Having reviewed these studies, Hayward et al. (2017) developed and validated a brief form of CRI with a sample of adults from the United States (*n* = 1,391), which includes 10 items loading on two subscales of reappraisal and Disengagement coping. Reappraisal coping is an adaptive coping mechanism correlated with higher welfare, while Disengagement coping echoes a maladaptive sort of coping connected with poorer welfare. Reappraisal subscale items echo the positive reappraisal of stigmatizing situations. This subscale yielded significant positive correlations with its counterpart subscale in Brief COPE and the ways of coping measure, lower levels of internalized weight bias, body form worries, depression, anxiety and stress, and elevated self-esteem. At the same time, it was negatively related to self-blame, behavioral Disengagement, and escape-avoidance. On the other hand, the disengagement subscale items measure several adverse reactions to weight stigma, including withdrawal, pessimistic self-talk, and departure from circumstances due to the fear of experiencing additional stigma. It displayed positive associations with the avoidance, Disengagement, and self-blame subscales of general coping measures, and poorer psychological outcomes such as more significant internalized weight bias, body form concerns, body dissatisfaction, depression, anxiety and stress, and lower self-esteem and negative associations with the active coping and positive reframing scores (Hayward et al., 2017). Overall, this brief form of CRI (BCRI) provides a practical tool to assess coping with weight stigma and may encourage researchers to advance the literature on the outcomes of weight stigma. However, research on the BCRI is still early in the validation process, as no other studies have yet examined the psychometrics of the measure to our knowledge, making it uncertain to what extent findings can be generalized to other societies and age groups. Therefore, more work is needed to further validate the BCRI with samples from other cultures (e.g., Eastern Cultures) and age groups (e.g., adolescents). Also, it is yet to be studied whether the factor structure of the BCRI is invariant across gender groups; the establishment of measurement invariance (MI) indicates that a consistent factor structure underlies the measure across groups, permitting mean comparisons among groups (Han et al., 2019).

In an effort to address these limitations, this study examined the psychometric properties of the Persian version of BCRI with a sample of mixed gender Iranian school-attending youth. First, to examine the construct validity of the Persian BCRI, we will conduct a confirmatory factor analysis (CFA) to examine the originally proposed two-factor model of the BCRI. Next, this study will be the first to study whether the factor structure of the Persian BCRI is invariant across gender groups. Third, to examine the internal consistency of the Persian BCRI subscales scores, the McDonald Omega coefficients and Chronbach’s α values will be calculated. Finally, to test the convergent and divergent validity of the BCRI scores, we examined their associations with external correlates of interests (e.g., anxiety, depression, body dissatisfaction, confrontative, and escape-avoidance coping strategies). More specifically, based on theory and prior research, we expected the Reappraisal coping to be positively related to confrontative and positive reappraisal and negatively with anxiety, depression, and body dissatisfaction, while the Disengagement subscale was hypothesized to yield positive associations with anxiety, depression, stress, and escape-avoidance coping strategy, while it would demonstrate negative associations with confrontative and positive reappraisal coping strategies.

## Materials and methods

### Participants and procedure

This study was first approved by the participating schools and the ethics committee of the Iran University of Medical Sciences (code number = IR.IUMS.REC.1400.458). A total of 832 school-attending 15–19 years-old youth (M age = 15.23; *SD* = 1.83; 49.60% boys) were recruited online from schools in Tehran from May 2021 to September 2021. The students and their parents were first contacted using a secured online platform (Shad application) and were informed about the aims and the voluntary and confidential character of the study. Students enrolled in the study and completed the measures if their parents and they themselves provided online signed informed consent. Students anonymously completed the questionnaires online in a standardized order using a secured online platform at a time and location of their convenience (due to the COVID-19 pandemic, the schools were closed at the time of the data collection). Of the 832 participants, 253 (*M_age_* = 15.38, *SD* ± 0.50; 53% female) respondents had a BMI score of >30, and all the analyses were computed based on the data from these 253 participants. Inclusion criteria consisted of having an interest in completing questionnaires and being a school-attending student; exclusion criteria included having a diagnosis of severe psychiatric disorder during life, a history of being hospitalized in a psychiatric hospital, and the use of psychiatric medications (all of which were assessed *via* self-report information).

## Measures

### Brief coping responses inventory

BCRI (Hayward et al., 2017) is the short form of the Coping Responses Inventory (CRI) and measures various coping approaches in reaction to weight stigma. The measure includes ten items rated on a Likert-type scale ranging from 0 (*Never*) to 9 (*Daily*) and load on two subscales of Reappraisal (5 items) and Disengagement (5 items). The essence and psychometric properties of the CRI have been reviewed previously.

*Persian BCRI*. For the present study, the original BCRI was first translated to Persian by two translators eloquent in English. Afterward, a third independent translator back-translated Persian translations to English. Next, the back-translated English version of the BCRI was examined and reviewed by the authors. If needed, revisions were made to the items in order to make them as clear as possible (Epstein et al., 2015).

### Body mass index

To include participants with a BMI score of <30, we calculated the BMI scores of the participants based on the self-reported data (i.e., participants’ weight and height) and according to the following formula: BMI = Weight (kg)/Height (m^2^).

### Depression anxiety stress scale-21

DASS-21 (Lovibond and Lovibond, 1995) is a self-report measure with 21 items and three subscales of depression, anxiety, and stress (7 items per subscale). Items are scored on a Likert-type scale ranging from 0 (*did not apply to me at all*) to 3 (a*pplied to me very much*). Subscale scores are computed by summing up the items scores of each subscale. The Persian version of the DASS-21 yielded adequate psychometric properties (Sahebi et al., 2005).

### Ways of coping questionnaire

WCQ assesses cognitions and behaviors individuals utilize to handle internal or external needs in specific stressful encounters. It consists of 66 items, rated on a 4-point Likert-type scale ranging from 0 (*does not apply and/or not used*) to 3 (*used a great deal*). WCQ consists of the following eight factors: Confrontive Coping, Distancing, Self-Control, Seeking Social Support, Accepting Responsibility, Escape Avoidance, Planful Problem Solving, and Positive Reappraisal (Folkman and Lazarus, 1985, 1986). In this study, we only administered items belonging to Confrontive, Escape Avoidance, and Positive Reappraisal Coping Styles. The Persian version of WCQ was found to have adequate psychometric properties (Padyab et al., 2012).

### Body dissatisfaction measure

To measure body dissatisfaction, we used the Body Dissatisfaction Subscale of the EDI-III (Garner, 2004), which was developed to measure dissatisfaction with diverse body areas. The subscale includes ten items rated on a Likert-type scale ranging from “*never*” to “*always*.” Following the recommendations of Garner (2004), we converted the original item scores to a five-point scale (never and rarely responses = 0; sometimes – always = 1 through 4). The Persian version of the EDI-III yielded promising results with a sample of students in Iran (Dadgostar et al., 2017).

### Data analyses

Following the analyses strategy in prior psychometric works in Iran (Ebrahimi et al., 2021, 2022; Taheri et al., 2021; Elhami Athar et al., 2022a,b), using the JASP free software (2021), we first examined the construct validity of the Persian BCRI by performing confirmatory factor analysis (CFA) on the two-factor model of BCRI with the Diagonally Weighted Least Squares (DWLS) estimator, which is appropriate for ordinal data (Flora and Curran, 2004). Model fit was evaluated based on the Tucker–Lewis index (TLI), the comparative fit index (CFI), and the root mean square error of approximation (RMSEA). RMSEA scores below 0.08 and a TLI/CFI scores of 0.90 or more were considered to indicate a good fit (Bentler, 1990; Hu and Bentler, 1999). Next, measurement invariance (MI) was performed across gender groups according to the sequential strategy suggested by Meredith and Teresi (2006). We first tested the two-factor model individually for boys and girls samples. Then, three levels of MI (i.e., configural, metric, and scalar) were tested to examine whether the factor structure, factor loadings, and item intercepts, respectively, were invariant across gender groups. According to Cheung and Rensvold (2002), change in CFI (ΔCFI), i.e., ΔCFI smaller than or equal to 0.01, was considered to support MI. If MI analyses is supported, we will use Student’s *t*-test to examine if significant gender differences exist for the BCRI scores, while we also examine Cohen’s *d* as a measure of effect size (Cohen, 2013).

Third, we examined the internal consistency of the BCRI scores using McDonald Omega coefficient (*ω*) and Cronbach’s alpha (*α*), which is defined as low (≤0.59), marginal (0.60–0.69), acceptable (0.70–0.79), good (0.80–0.89), and excellent (≥0.90; Cheung and Rensvold, 2002). We also used mean inter-item correlation (MIC) scores as another measure of internal consistency, with values ranging from 0.15 to 0.50 being considered adequate (Clark and Watson, 1995). Finally, associations between the BCRI scores and the external criterion variables were computed to examine the convergent and divergent validity of the BCRI scores (Cohen, 2013). A value of *p* of <0.05 was considered as the indicator of statistical significance. All analyses were conducted in SPSS 20.

## Results

### Confirmatory factor analyses

**Figure 1:**
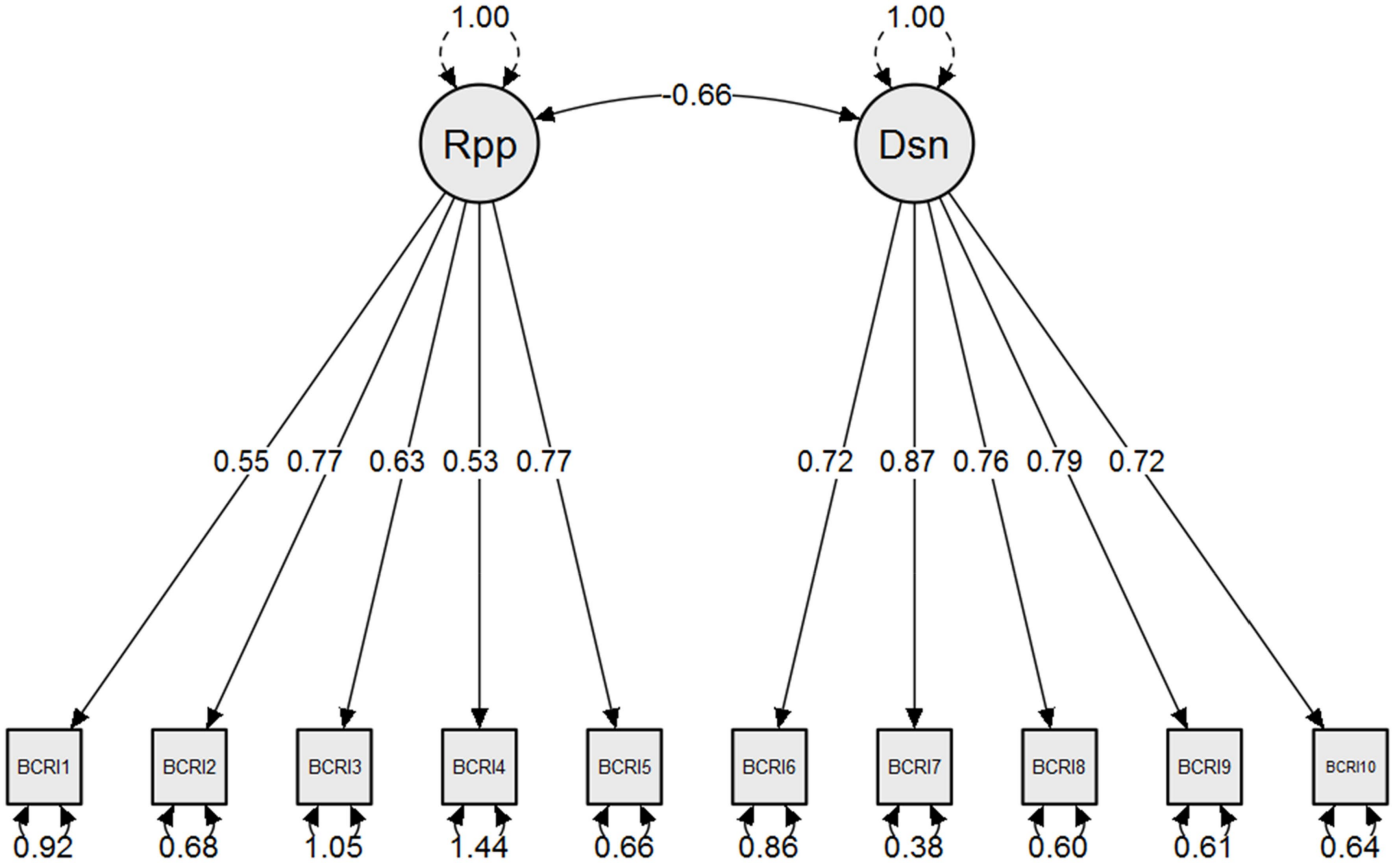
Standardized model parameters for the confirmatory factor analysis: two-factor model. Rpp, reappraisal; Dsn, disengagement.

The results of confirmatory factor analysis showed that the originally proposed two-factor model of the BCRI yielded excellent fit in the total (RMSEA = 0.028; CFI = 0.996; TLI = 0.994), boys (RMSEA = 0.026; CFI = 0.995; TLI = 0.993), and girls (RMSEA = 0.029; CFI = 0.997; TLI = 0.995) samples (Table 1). The standardized item loadings for this factor structure in the total sample could be retrieved from Table 2; Figure 1. Further analyses showed that this two-factor model is invariant across gender groups based on Configural (RMSEA = 0.001; CFI = 0.997; TLI = 0.997), Metric (RMSEA = 0.001; CFI = 0.997; TLI = 0.996), and Scalar (RMSEA = 0.001; CFI = 0.996; TLI = 0.996) invariances, which enables gender comparisons possible.

**Table 1:** The Goodness of Fit indices for the two-factor model of BCRI in the total and subsamples of girls and boys.

	Fit indices results			Critical values		
Sample	CFI	TLI	RMSEA	CFI	TLI	RMSEA
Total sample (*n* = 256)	0.996	0.994	0.028	0.90	0.90	0.08
Boys (*n* = 109)	0.995	0.993	0.026	0.90	0.90	0.08
Girls (*n* = 147)	0.997	0.995	0.027	0.90	0.90	0.08

CFI, comparative fit index; TLI, Tucker–Lewis index; RMSEA, root mean square error of approximation; CFA, confirmatory factor analysis.

**Table 2:** Standardized item loadings for the two-factor model of the BCRI (*n* = 253).

Subscale	Item	Item content	Std. loading
Reappraisal	1	I try to think about good things that have happened to me	0.547
	2	I remind myself that I am a good person and people like me just the way I am	0.771
	3	If someone has a problem with how I look, I see it as their problem, not mine.	0.628
	4	If people do not like me because of my size, I see it as their loss, not mine.	0.533
	5	I love myself, even when it seems like other people do not.	0.773
Disengagement	6	I feel really bad about myself.	0.722
	7	I get depressed and isolate myself.	0.870
	8	I avoid looking in the mirror so that I do not have to think about my weight.	0.756
	9	I think that no one will ever love me because of my weight.	0.788
	10	I avoid going out in public because I am afraid people will make comments about my size.	0.717

BCRI, brief coping responses inventory; Std. loading, standardized loading. All loadings were statistically significant at *p* < 0.001.

### Descriptive information and internal consistency

As shown in Table 3, when using the McDonald Omega coefficient and Chronbach’s alpha as measures of reliability, the internal consistency for the BCRI subscales of reappraisal and Disengagement was in the good range in the total sample and across gender groups. The results also yielded significant mean differences in only the disengagement subscale of the BCRI [*t* (254) = 3.94, *p* = 0.001, *d* = 0.48] in which girls scored significantly higher than boys (Table 3).

**Table 3:** Mean, standard deviation, and internal consistency of the BCRI in the total sample and in subsamples of boys and girls separately.

Measures	Total sample (*n* = 256)				Girls (*n* = 147)	Boys (*n* = 109)	Gender group comparison^a^	
	M (*SD*)	*α*	ω	MIC	M (*SD*)	M (*SD*)	*p*	*d*
BCRI								
Reappraisal	12.51 (4.75)	0.79	0.79	0.42	12.23 (4.82)	12.89 (4.66)	0.27	0.14
Disengagement	5.22 (5.08)	0.88	0.88	0.53	6.23 (5.51	3.86 (4.09)	0.01	0.48
DASS-21								
Depression	6.29 (5.25)	0.78	0.79	0.38	7.26 (5.11)	4.99 (4.59)	–	–
Anxiety	4.77 (4.29)	0.76	0.76	0.36	5.81 (4.53)	3.37 (3.52)	–	–
Stress	7.06 (4.86)	0.79	0.80	0.41	8.31 (4.92)	5.39 (4.26)	–	–
WCQ								
Confrontive coping style	7.14 (3.11)	0.68	0.68	0.31	7.02 (3.14)	7.23 (3.09)	–	–
Escape avoidance coping style	8.05 (4.33)	0.71	0.71	0.42	8.66 (4.33)	7.23 (4.21)	–	–
Positive reappraisal coping style	11.31 (4.44)	0.76	0.77	0.39	11.14 (4.36)	11.55 (4.59)	–	–
Body dissatisfaction	14.41 (6.76)	0.83	0.83	0.49	15.50 (6.66)	12.96 (6.64)	–	–

BCRI, brief coping responses inventory; DASS-21, the depression, anxiety and stress scale – 21 items; WCQ, ways of coping questionnaire; M, mean; SD, standard deviation; ω, McDonald omega coefficient; MIC, mean inter-item correlation; *d*, Cohen’s *d*. ^a^Since no MI analyses have been done with Persian DASS-21 and WCQ, we did not conduct gender comparisons on scores from these measures.

### Convergent/divergent validity

In support of their convergent/divergent validity, the BCRI Reappraisal subscale was positively and significantly associated with confrontive and positive reappraisal coping styles scores, while it was negatively associated with depression, anxiety, stress, and escape avoidance coping style, and body dissatisfaction score. On the other hand, the BCRI Disengagement subscale demonstrated significant positive associations with anxiety, depression, stress, body dissatisfaction, and escape avoidance coping style scores; at the same time, it yielded a significant negative association with positive reappraisal coping style scores (Table 4). Findings were substantially similar in the boys and girls subsamples, though with two exceptions: BCRI Reappraisal was significantly and positively associated with confrontive coping style only in the boys subsample, while it yielded a significant negative association with escape avoidance coping style only in the girls subsample (Table 5).

**Table 4:** Pearson correlation coefficients between bcri subscales scores and other study variables (*n* = 256).

	Reappraisal	Disengagement
Reappraisal	–	–
Disengagement	−0.54**	–
Depression	−0.39**	0.54**
Anxiety	−0.33**	0.53**
Stress	−0.36**	0.49**
Confrontive coping style	0.21**	0.01
Escape avoidance coping style	−0.18**	0.48**
Positive reappraisal coping style	0.43**	−0.23**
Body dissatisfaction	−0.38**	0.53**

BCRI, brief coping responses inventory. **p < 0.001; *p < 0.05.

**Table 5:** Pearson correlation coefficients between bcri subscales scores and other study variables for subsamples of girls and boys.

Measure	Gender			
	Girls (*n* = 147)		Boys (*n* = 109)	
	Reappraisal	Disengagement	Reappraisal	Disengagement
Reappraisal	–	–	–	–
Disengagement	−0.65**	–	−0.34**	–
Depression	−0.44**	0.53**	−0.28**	0.50**
Anxiety	−0.41**	0.49**	−0.34**	0.51**
Stress	−0.44**	0.50**	−0.21*	0.35**
Confrontive coping style	0.09	0.10	0.38**	−0.11
Escape avoidance coping style	−0.31**	0.45**	0.03	0.49**
Positive reappraisal coping style	0.42**	−0.25**	0.45**	−0.24**
Body dissatisfaction	−0.47**	0.54**	−0.25**	0.46**

BCRI, brief coping responses inventory. **p < 0.001; *p < 0.05.

## Discussion

In the current study, we aimed to examine the factor structure, reliability, and convergent/divergent validity of the Persian version of the BCRI with a sample of school-attending youth in Iran. We first aimed to test the originally proposed two-factor model of the BCRI (Hayward et al., 2017). The confirmatory factor analysis results indicated that this model reached excellent model fit. This study is also the first to examine and report that the measurement invariance for the BCRI scores was in the excellent range across gender groups. Strong MI results indicate a consistent underlying structure across groups and allow for mean comparison of the BCRI scores across genders (Han et al., 2019). In this vein, our results showed that girls scored significantly higher in the disengagement subscale of the BCRI, indicating that women react to weight stigma significantly higher than men with negative reactions (e.g., withdrawal, pessimistic self-talk, and avoidance of some situations due to the fear of experiencing additional stigma), which is consistent with the results of Sattler et al. (2018) that males and females are not affected similarly by weight-stigma experiences. Specifically, Sattler et al. (2018) found that females reported significantly more weight stigma experiences than males and that higher stigma experiences are associated with higher levels of walking and vigorous physical activity in men, while for women, frequent weight stigma experiences were associated with lower autonomous motivation and lower levels of physical activity. Studies also indicate that males demonstrate less internalization of weight bias than females, suggesting that variations in internalization of weight bias across genders result in different coping strategies (Pearl et al., 2014; Boswell and White, 2015; Sattler et al., 2018).

Furthermore, the BCRI subscales scores yielded acceptable to good internal consistency based on the McDonald Omega coefficient, Cronbach’s alpha, and MIC values. Thus, it can be concluded that the Persian BCRI scores are internally consistent. Finally, the current study also examined correlations between BCRI scores and external criterion measures to bolster what is known about the convergent/divergent validity of the Persian version of this self-report tool. As expected based on theory and prior studies, the BCRI reappraisal and Disengagement subscales scores demonstrated the hypothesized relations with external correlates of interests (e.g., anxiety, depression, body dissatisfaction, and escape/avoidance coping style). Indeed, reappraisal is a positive coping in which stressful events are re-construed as benign, beneficial, and meaningful (Garland et al., 2011). Thus, individuals who implement this strategy are expected to be less depressed and anxious and have higher self-esteem levels, as our results demonstrated. On the other hand, disengagement strategy includes attempts to keep away from or minimize stressful events and is negatively correlated with psychological well-being (Bourguignon et al., 2020); this was further supported according to our results. Overall, our results are in line with the original study indicating that reappraisal coping is an adaptive coping strategy and is associated with higher well-being and self-esteem and lower levels of depression, anxiety, internalized weight bias, and body shape concerns, while disengagement coping mirrors a maladaptive coping style and is associated with poorer welfare, escape-avoidance coping style, withdrawal, negative self-talk, the avoidance, body dissatisfaction, depression, anxiety stress, and lower self-esteem (Hayward et al., 2017). Conclusively, the results support the validity of the interpretation of BCRI scores. Our results suggested two significant gender differences in the correlation analyses. First, BCRI Reappraisal yielded a significantly positive association with confrontive coping style only in the boys subsample. As the confrontive coping style includes making aggressive efforts to change the situation to the point of being risky and antagonistic, it is estimated that due to the gender role and physiological differences across the genders, males cling to the confrontive coping style significantly more than females. Second, BCRI Reappraisal was significantly and negatively correlated with escape avoidance coping style only in the girls subsample. This also makes sense when considering the previous finding. It seems that among males, the use of Reappraisal coping in stigma situations is not related to the extent to which they use escape avoidance coping style. However, since our subsamples were small, the findings concerning the gender differences are in need for replication and should be interpreted with caution.

Our findings should be interpreted considering a few limitations. First, we used self-report measures to gather data and to examine the convergent/divergent validity of the BCRI scores. Thus, shared method variance might partly explain the associations of BCRI scores with external correlates of interests. Second, our study sample included only school-attending youth who were recruited through a convenience sampling method, so the results should not be generalized to other groups. Third, due to the limitations caused by the COVID-19 pandemic, we used self-report data to calculate BMI scores which could have influenced the results. Despite these limitations, our results indicated that the Persian BCRI has a solid factor structure that is invariant across genders, enjoys acceptable internal consistency, and is associated with external correlates of interest in line with theory and prior research. The BCRI provides a swift and efficient way to measure core coping responses to weight stigma experiences. Our findings may encourage research on weight-related stigma experiences in other Iranian settings (e.g., community, university students, and clinical samples). Finally, our results have some clinical implications for the practitioners. First, some individuals with obesity do not seek professional help or leave the therapy sessions at all because they think that the mental health professionals will stigmatize them as well, so they implement the disengagement way of dealing with the problem. Therapists could use the BCRI to examine how individuals deal with the stigma experiences. Individuals with higher levels of sensitivity to stigma might need a more considerate and empathetic therapist. Second, the pattern of reactions to the weight stigma could reflect an individual’s overall pattern of dealing with non-weight-related stigmas, which could be informative for the therapists in therapeutic sessions.

## Data availability statement

The raw data supporting the conclusions of this article will be made available by the authors, without undue reservation.

## Ethics Statement

The studies involving human participants were reviewed and approved by Research Deputy of Iran University of Medical Sciences. Written informed consent to participate in this study was provided by the participants’ legal guardian/next of kin.

## Author contributions

LP: gathered the data, performed data analyses, and prepared the manuscript. AF, FL, HF, and BG: reviewed and revised the manuscript. All authors have contributed to the study and agreed to the publication of the manuscript.

## Conflict of interest

The authors declare that the research was conducted in the absence of any commercial or financial relationships that could be construed as a potential conflict of interest.

## Publisher’s note

All claims expressed in this article are solely those of the authors and do not necessarily represent those of their affiliated organizations, or those of the publisher, the editors and the reviewers. Any product that may be evaluated in this article, or claim that may be made by its manufacturer, is not guaranteed or endorsed by the publisher.
